# Genetically confirmed coexistence of neurofibromatosis type 1 and Cherubism in a pediatric patient

**DOI:** 10.1007/s11033-024-09214-0

**Published:** 2024-01-28

**Authors:** Sofia Sarantou, Nikolaos M. Marinakis, Joanne Traeger-Synodinos, Ekaterini Siomou, Argyrios Ntinopoulos, Anastasios Serbis

**Affiliations:** 1 Medical School, University of Ioannina, Ioannina, Greece; 2 Laboratory of Medical Genetics, St. Sophia’s Children’s Hospital, National and Kapodistrian University of Athens, Athens, Greece; 3 Department of Pediatrics, School of Medicine, University of Ioannina, St. Niarchos Av, Ioannina, 45100 Greece

**Keywords:** Neurofibromatosis, NF1, Cherubism, RASopathy

## Abstract

**Background:**

Neurofibromatosis type 1 (NF1) is an autosomal dominant disorder typified by various combination of numerous Café-au-lait macules, cutaneous and plexiform neurofibromas, freckling of inguinal or axillary region, optic glioma, Lisch nodules and osseous lesions. Cherubism is a rare genetic syndrome described by progressive swelling of the lower and/or upper jaw due to replacement of bone by fibrous connective tissue. Patients are reported in the literature with NF1 and cherubism-like phenotype due to the NF1 osseous lesions in the jaws. The purpose of this case report is the description of a young male genetically diagnosed with both NF1 and cherubism.

**Methods and results:**

A 9 years and six month old patient with clinical findings of NF1 and cherubism in whom both diseases were genetically confirmed, is presented. The patient was evaluated by a pediatrician, a pediatric endocrinologist, an ophthalmologist, and an oral and maxillofacial surgeon. A laboratory and hormonal screening, a histological examination, a chest X-ray, a magnetic resonance imaging (MRI) of the orbit and a digital panoramic radiography were performed. Genetic testing applying Whole Exome Sequencing was conducted.

**Conclusions:**

A novel and an already reported pathogenic variants were detected in *NF1* and *SH3BP2* genes, respectively. This is the first described patient with coexistence of NF1 and cherubism. The contribution of Next Generation Sequencing (NGS) in gene variant identification as well as the importance of close collaboration between laboratory scientists and clinicians, is highlighted. Both are essential for optimizing the diagnostic approach of patients with a complex phenotype.

## Introduction

Neurofibromatosis type 1 (NF1) is a clinically diverse, neurocutaneous hereditary syndrome that affects approximately 1:2600 to 1:3000 individuals [1]. NF1 is caused by mutations in the tumor suppressor neurofibromin 1 -*NF1*- gene [2], which codes for a protein that regulates the RAS signalling pathway [3]. Clinical features of NF1 are highly variable and include multiple café-au-lait macules (CALMs), Lisch nodules in the iris of the eyes, neurofibromas in the skin, plexiform neurofibromas (PNFs), and osseous defects. On the other hand, Cherubism is an uncommon inherited syndrome characterized by progressive bilateral enlargement of the mandible and/or maxilla and it is caused by mutations in the *SH3PB2* gene (4p16.3) [4].

Cherubism’s characteristic facial phenotype is often clinically indistinguishable from NF1, because both are associated with osseous tissue malformations. Although, there are patients reported with NF1 and cherubism-like phenotype, there is no literature report with a genetically proven co-existence of both genetic diseases [5–7]. According to our knowledge, this case report describes the first pediatric patient with mutations in both *NF1* and *SH3PB2* genes and clinical manifestations of both diseases.

## Case presentation

**Fig. 1:**
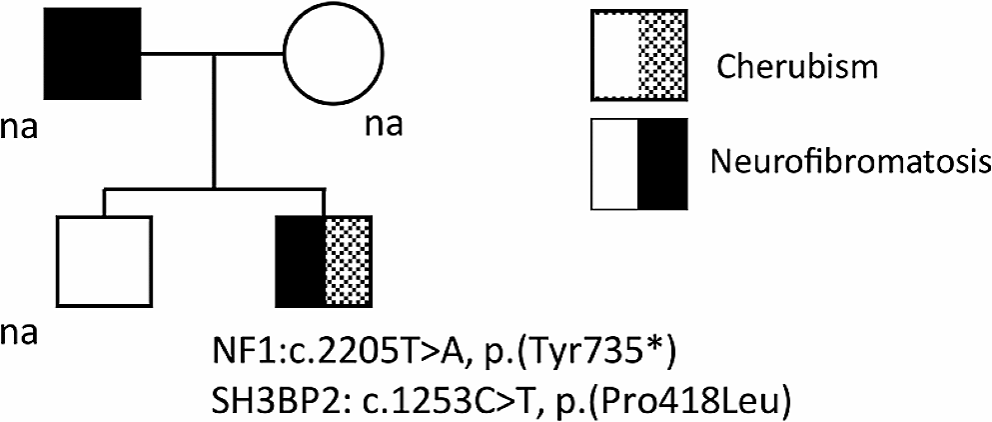
Pedigree of the family and diagnosis results of the proband. na: not available for genetic testing

A 9 years and six month old male presented to our medical center with his family for consultation regarding facial dysmorphism. Medical history records were significant of dysplasia of the upper and lower jaw which started at the age of 5 years of age with a slow gradual progression over time. A NF1 clinical phenotype was reported in his father with mild clinical features, but, unfortunately, genetic testing cannot be conducted, as he was killed in a car accident. No other relevant family history of jawbone or maxilla enlargement was reported (Fig. 1). Clinical examination of our patient revealed NF1-related manifestations, including multiple CALMs (eleven with a diameter > 5 mm) and thoracic and lumbar spine scoliosis. Lisch nodules and cutaneous/dermal neurofibromas (CNFs) were absent. The examination was also significant for full round cheeks and upward cast of the eyes, possibly due to the progressive painless bilateral enlargement of both the lower and the upper jaws, creating the characteristic “cherubic” appearance. Moreover, abnormal dentition with alteration in teeth shape, displaced roots and increased tooth mobility and instability were identified. Annual growth rate of the patient was borderline since infancy, growing along the 3rd percentile for height and having a height of 125.8 cm at 9^6/12^ years. His weight was 30 kg and his body mass index (BMI) 18,5 kg/m^2^ (85th percentile) and he reported a low weight gain rate over the last few years. Laboratory tests for follicle-stimulating hormone (FSH), luteinizing hormone (LH), testosterone, DHEA sulfate (DHEAS), 17-0 H progesterone, adrenocorticotropic hormone (ACTH), cortisol, prolactin, thyroid stimulating hormone (TSH), free thyroxine (fT4), insulin-like growth factor-1 (IGF-1), parathyroid hormone (PTH), 25-hydroxy vitamin D (25(OH)D), 1,25-dihydroxy vitamin D (1.25(OH)_2_D), calcium, and phosphate, tissue transglutaminase IgA antibody (tTG IgA) for celiac disease and ferritin, were performed with no abnormal results.

Computer tomography (CT) of the visceral cranium and Magnetic resonance imaging (MRI) of the orbit and the brain revealed osteolytic lesions with increased dimensions of intraosseous formations in the maxilla, mandible, and both zygomatic bones. Diffusion weighted imaging (DWI) showed no diffusion inside the osteolytic lesions, supporting the diagnosis of benign malformations. Additionally, MRI showed multiple hamartomatous lesions on both the left and the right cerebellar peduncles, the posterior aspect of the putamen, both the hypothalami, and the left thalamus. Furthermore, a cisterna magna malformation of the posterior fossa and bilateral cervical reactive lymphadenopathy were noticed.

A histological examination of the maxillary and mandibular lesions was carried out. Two white-yellow (max diameter 0.9 cm, 0.7 cm) fragments and, two red-gray pieces of tissue of soft consistency were received (m.d. 0.5 cm, 0.7 cm). The biopsy identified a central giant cell granuloma. Histologic features included multinucleated giant cells of osteoclastic type, which were scattered within richly vascularized myofibroblastic and fibrous connective tissue with hemorrhagic spots. The myofibroblastic stromal element consisted of bundles of interlaced ovoid or spindle-shaped mononuclear cells, fusion of which resulted in formation of syncytia with multinucleated osteoclastic cells. Furthermore, proteinaceous eosinophilic depositions were distributed throughout the perivascular space. Lastly, immunohistochemical analysis was conducted. Calponin, smooth muscle actin (SMA), CD68 KP1, CD163 and leukocyte common antigen (LCA) were expressed. In conclusion, the above-mentioned findings support the diagnosis of cherubism.

### NF1 diagnosis

The possible diagnosis of NF1 in our patient was examined on the basis of the most recently updated (2021) diagnostic criteria for NF1 [8]. The only positive criterion for our patient was the presence of 11 CALMs with a diameter > 5 mm. His father had a similar clinical appearance suggestive of NF1, namely multiple CALMs, but he had not been genetically tested. Therefore, according to the relevant criteria, the clinical diagnosis of NF1 in the proband could not be established. Nevertheless, our clinical assumption of NF1 was strong enough to proceed with a genetic test, which confirmed our hypothesis.

### Cherubism diagnosis

Cherubism diagnosis is clinically established based on age, family history, typical clinical, radiographic, and histologic findings. This 9 years and six month old male with no relevant family history of cherubism appeared with bilateral dysplasia of maxilla and mandible which corresponded to osteolytic lesions on CT and MRI, central giant cell granuloma on histological examination and positively expressed specific immunohistochemical agents.

### Differential diagnosis

More than 100 genetic conditions that include CALMs or other NF1 features have been described. In Table 1, the most relevant conditions, and associated genes of interest in the differential diagnosis of NF1 are summarized [3].

**Table 1:** Details about the syndromes included in the differential diagnosis

Disorder	Mode of inheritance	Chromosome location	Gene(s)	Clinical features
Neurofibromatosis 1 (NF1)	Autosomal Dominant	17q11.2	*NF1 tumor suppression gene*	– Café-au-lait macules Freckling in the axillary or inguinal regions – Neurofibromas – Plexiform neurofibromas – Optic pathway glioma – Lisch nodules – Osseous lesions
Cherubism		4p16.3	*SH3BP2*	– Bilateral enlargement of mandible & maxilla - “cherubic habitus” – Central giant cell granuloma with osteoclastic type & proteinaceous perivascular eosinophilic depositions
Noonan syndrome (NS)	Autosomal Dominant		*PTPN11, SOS1, SOS2, RAF1, KRAS, NRAS, MRAS, MAP2K1, BRAF, RASA2, LZTR1, RRAS, RIT1*	– Craniofacial dysmorphisms – Broad or webbed neck – Short stature – Congenital heart defects – Coagulopathies – Osseous deformities – Developmental delay
Noonan syndrome with multiple lentigines (NSML or LEOPARD syndrome)	Autosomal Dominant		*BRAF, MAP2K1, PTPN11, and RAF1*	– Lentigines – Craniofacial dysmorphisms (widely spaced eyes and ptosis) – Cardiac abnormalities – Sensorineural hearing loss – Short stature – Developmental delay
Cardiofaciocutaneous (CFC) syndrome	Autosomal Dominant		*BRAF, MAP2K1, MAP2K2, KRAS*	– Craniofacial dysmorphisms – Congenital heart disease and rhythm disturbance – Dermatological abnormalities (xerosis cutis, sparse/curly/ brittle hair, dystrophic nails) – Neurological manifestations (hypotonia, seizures) – Developmental delay – Cognitive impairment (mild to severe)
Ramon syndrome (OMIM 266,270)	Autosomal Recessive	Unknown	*Unknown*	– Bilateral painless swelling of the mandible and maxilla - “cherubic habitus” – Gingival fibromatosis – Epilepsy – Intellectual disability
Hyperparathyroidism-jaw tumor (HPT-JT) syndrome	Autosomal Dominant	1q31.2	*CDC73*	– Benign ossifying fibromas in the mandible, maxilla, kidneys & uterus – Parathyroid adenomas – Hyperparathyroidism – Hypercalcemia
Familial isolated hyperparathyroidism (FIHP, HRPT1)	Autosomal Dominant	1q31.2	*CDC73*	– Benign fibro-osseous giant cell lesions (brown tumors) in mandible & maxilla – Hyperparathyroidism – Hypercalcemia
Fibrous dysplasia/McCune-Albright syndrome (FD/MAS)	sporadically occurring	20q13.32	*GNAS*	– Polyostotic fibrous dysplasia – Café-au-lait macules – Peripheral precocious puberty – Ground-glass appearance on radiography
Central giant-cell granuloma	Unknown	Unknown		– Benign uniocular lesions of mandible and maxilla – Central giant cell granuloma

### Genetic testing

**Fig. 2:**
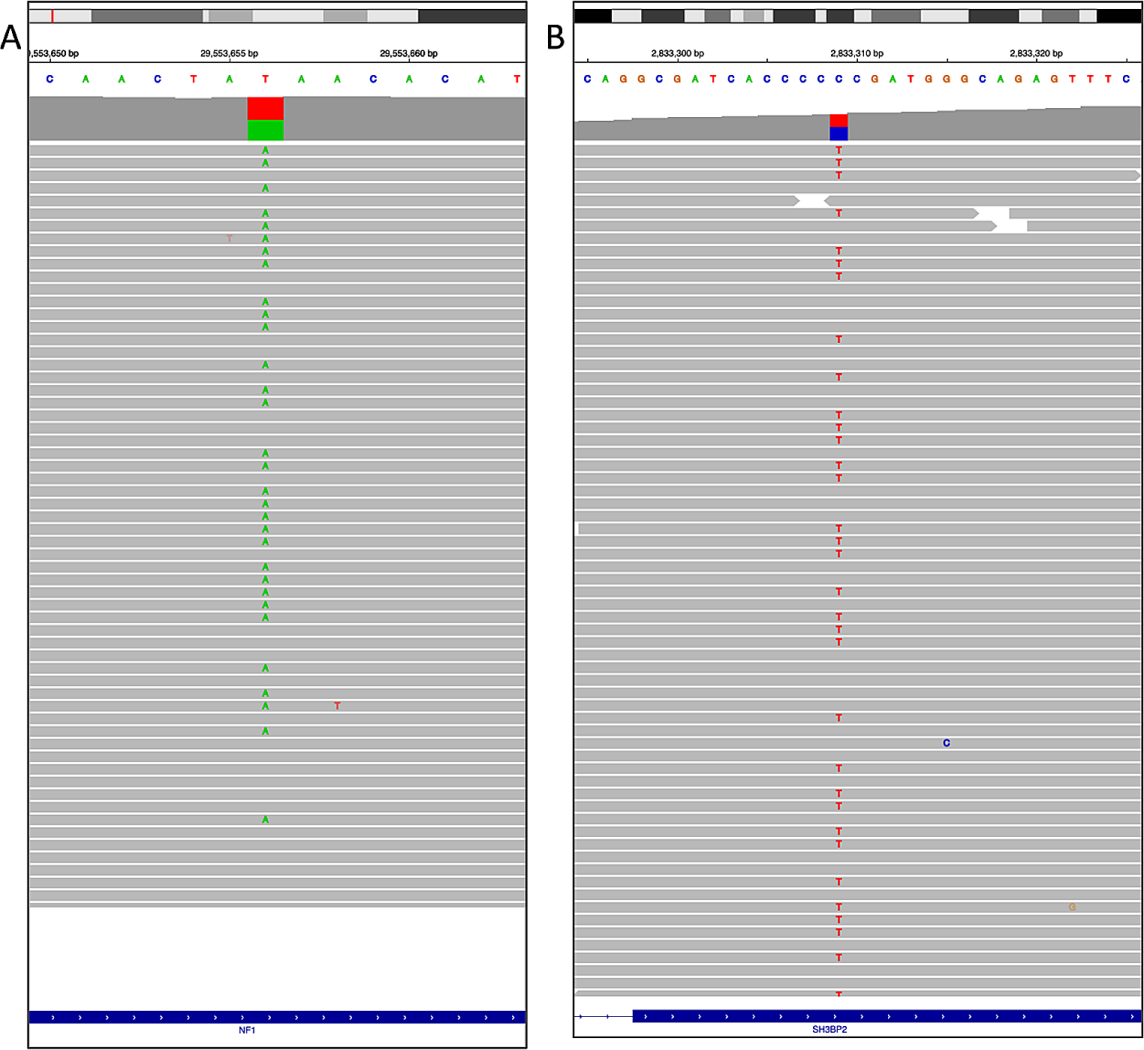
IGV screenshots of the proband’s WES, **A** *NF1* gene variant c.2205T > A, **B** *SH3BP2* gene variant c.1253 C > T

**Fig. 3:**
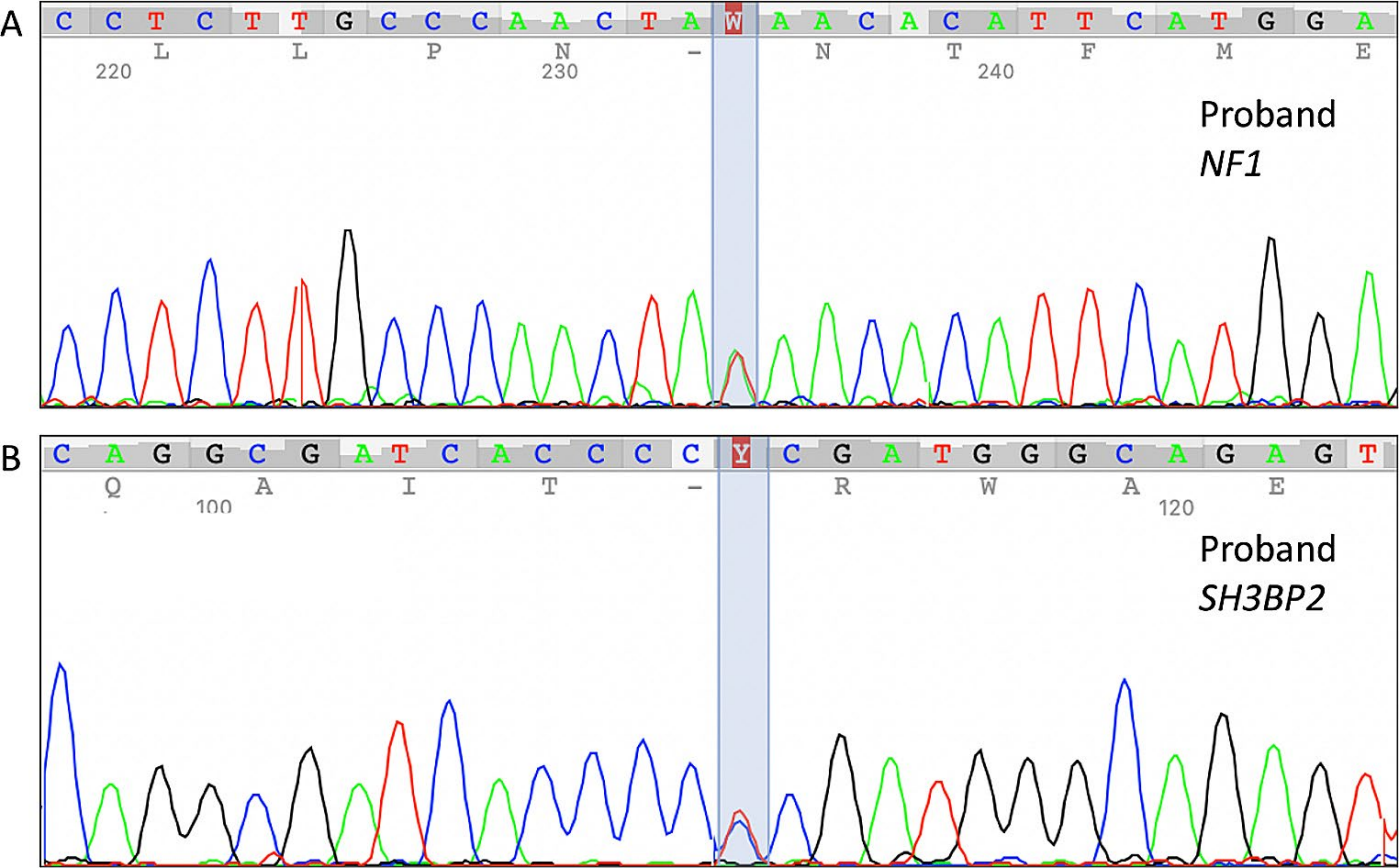
Partial Sanger sequencing chromatographs of the proband, **A** *NF1* gene variant c.2205T > A, **B** *SH3BP2* gene variant c.1253 C > T

Ethical approval for the genetic study was acquired from St. Sophia Children’s Hospital Scientific and Ethics Committee (Approval number: 3669/12-02-18). All patient data were processed and stored according to the guidelines of the General Data Protection Regulation (GDPR). Written informed consent was provided by the patient’s parents. Following pre-test counselling proband only-WES (~ 20,000 genes) was performed, using genomic DNA extracted from whole blood. Library preparation was implemented using IDT xGen Exome Research v2 kit (Integrated DNA Technologies). The resulting libraries were subjected to paired-end sequencing on an Illumina NextSeq 500 platform. WES data from the bioinformatic analysis contained 102.138.898 number of reads and 25.169 variants (Fig. 2). The percentage of regions with at least 20X coverage was 98,5% and the mean coverage was 151X. Variant analysis was performed using VarSome Clinical platform [9] and was based on the phenotype-driven strategy [10]. For the variant classification, American College of Medical Genetics and Genomics (ACMG) guidelines were used [11]. Targeted Sanger sequencing for the specific variants in *NF1* and *SH3BP2* genes was performed for confirmation (Fig. 3).

Applying filter criteria based on phenotype-driven strategy [acceptance quality metrics, correlated phenotype using Human Phenotype Ontology terms (https://hpo.jax.org/app/), population frequency (GnomAD database, https://gnomad.broadinstitute.org), variant type, in-silico tools prediction, inheritance and allele state, variant classification), two heterozygous variants in different genes were detected. A novel nonsense variant NM_000267.3:c.2205T > A, p.(Tyr735*) in the *NF1* gene was detected, which is classified as pathogenic (PVS1, PM2, PP4) and an already reported missense variant NM_003023.4:c.1253 C > T, p.(Pro418Leu) in the *SH3BP2*, which is classified also as pathogenic (PM1, PM2, PM5, PP3, PP5) [12]. Unfortunately, family members for further genetic investigation were not available. In summary, the simultaneous occurrence of neurofibromatosis and cherubim were indisputably demonstrated.

## Discussion

To the best of our knowledge, this is the first report of the genetically verified coexistence of cherubism and NF1 in a pediatric patient. It underlines the important role of the genetic testing using NGS techniques and of the close collaboration between laboratory scientists and clinicians. ΝF1 diagnosis was clinically considered for our patient due to family history, but the imaging and histological findings revealed the co-existence of two different disorders.

Many nosological entities are characterized by mandibular and maxillary lesions. Cherubic habitus may be present in RASopathy syndromes, giant cell lesions of the jaws (GCLJ), hyperparathyroidism-jaw tumor (HPT-JT) syndrome, fibrous dysplasia/ McCune-Albright syndrome (FD/MAS), familial isolated hyperparathyroidism (OMIM 145,000), and Ramon syndrome (OMIM 266,270) [4] (Table 1).

NF1 belongs to the family of RASopathy disorders, arising from common genetic modifications in genes of the Ras/mitogen-activated protein kinase (MAPK) pathway [2, 13, 14]. Besides NF1, this group includes neurofibromatosis type 1–like syndrome (NFLS or Legius syndrome), Noonan syndrome (NS), Noonan syndrome with multiple lentigines (NSML or LEOPARD syndrome), Costello syndrome (CS), Cardio-Facio-Cutaneous (CFC) syndrome and Capillary Malformation–Arteriovenous Malformation syndrome (CM-AVM). Each of these possesses unique clinical features but many are common among the different syndromes such as, craniofacial dysmorphology, cardiovascular deformities, skeletal abnormalities, cutaneous lesions, neurocognitive impairment, and increased tumor risk. RASopathy syndromes may rarely appear simultaneously with Giant cell lesions of the jaws (GCLJ) which comprise an assembly of ailments depicted by deformities in the mandible and maxilla, concretely by multinucleated giant cells on histopathology examination [14]. Cherubism, Central giant cell granuloma, Brown tumor of hyperparathyroidism and Aneurysmal bone cyst are the main components of the GCLJ group [15].

NF1 is inherited in an autosomal dominant manner and is caused by loss-of-function variants in the *NF1* tumor suppression gene situated on 17q11.2. It is mainly branded by cutaneous neurofibromas, CALMS, optic gliomas, Lisch nodules and bone lesions. Osseous deformities including scoliosis, macrocephaly, and intraoral malformations can occur affecting soft tissues, dentition, mandible and maxilla [2]. Neurofibroma-induced facial abnormalities have not been reported in all patients and therefore, further investigation is necessary when NF1 patient presents with a facial abnormality. In this case report, the patient had bilateral enlargement of both the lower and the upper jaw and met the diagnostic criteria for NF1. Therefore, a CT scan and an MRI were preformed and revealed osteolytic lesions which were thought to be NF1 manifestations.

Regarding cherubic habitus, one of the main associated syndromes is Noonan syndrome (NS), a disorder of autosomal dominant inheritance. Characteristic findings include peculiar facial phenotype, short stature, a broad or webbed neck, congenital cardiac defects, coagulation complications, osseous deformities, and developmental impairment [6, 13–16]. Diagnosis of NS is established genetically by finding pathogenic variant in any of the following genes: *PTPN11, SOS1, SOS2, RAF1, KRAS, NRAS, MRAS, MAP2K1, BRAF, RASA2, LZTR1, RRAS* and *RIT1*. Noonan syndrome with multiple lentigines (NSML or LEOPARD syndrome), must also be included in the differential diagnosis. Cardinal features of NSML are lentigines, cardiac abnormalities (hypertrophic cardiomyopathy), diminutive stature, pectus excavatum and dysmorphic facies, comprising wide set eyes and ptosis [16]. Genetic testing is important since NSML is caused by mutations in *BRAF, MAP2K1, PTPN11*, and *RAF1*. Finally, a RASopathy syndrome, which should be considered in our case is Cardiofaciocutaneous (CFC) syndrome. CFC is characterized by dysmorphic craniofacial features, cardiac anomalies, neuromotor delay, cognitive impairment, ectodermal findings (xerosis cutis, sparse, curly and woolly or brittle hair, dystrophic nails), eye abnormalities (strabismus, nystagmus, and/or optic nerve hypoplasia) and hypotonia and is associated with alterations in *BRAF, MAP2K1, MAP2K2*, or *KRAS* genes [17]. As already mentioned, RASopathies share common clinical manifestations and overlapping mutated genes. NS, NSML and CFC were excluded from the possible diagnosis, as our patient tested negative for the corresponding genes.

The classic cherubic appearance is also a feature of Ramon syndrome. It is a rare autosomal recessive disorder characterized by gingival fibromatosis, bilateral painless swelling of the mandible and maxilla, epilepsy, intellectual disability, stunted growth, hypertrichosis, juvenile rheumatoid arthritis, and ocular abnormalities [18]. However, in our case, the absence of mental retardation, epilepsy, and gingival fibromatosis distinguishes cherubism from Ramon syndrome.

Hyperparathyroidism-jaw tumor syndrome (HPT-JT or HRPT2) and Familial isolated primary hyperparathyroidism (FIHP, HRPT1) are autosomal dominant disorders derived by a germline alteration of the cell division cycle 73 (CDC73) gene (1q31.2). This gene encodes for parafibromin which is a critical component of PAF1 complex that binds to RNA polymerase II and controls transcription [19]. Clinical manifestations of HPT-JT include highly elevated serum PTH levels, hypercalcemia, parathyroid adenomas and fibro-osseous tumors in the mandible and maxilla, kidneys and uterus. Jaw tumors are benign, bilateral or multifocal, enlarged, palpable or visible radiolucent mass lesions and fibro-osseous characterized by the presence of giant cells. FIHP is also characterized by increased levels of parathyroid hormone (PTH), calcium and alkaline phosphatase and is responsible for benign fibro-osseous giant cell lesions (brown tumors) in mandible and maxilla [20]. Both HPT-JT and FIHP can be distinguished from cherubism on the basis of increased levels of PTH, serum calcium, serum phosphorus and alkaline phosphatase. Our patient’s blood test results were within the normal range for the above-mentioned parameters and therefore both HPT-JT and FIHP were excluded from the differential diagnosis.

Another disease that should be included in the differential diagnosis is Fibrous dysplasia/McCune-Albright syndrome (FD/MAS). FD/MAS is a rare sporadically occurring syndrome derived by alteration in the *GNAS* gene (20q13.32) leading to excess production of cyclic AMP (cAMP) and therefore leading to proliferation and fibrosis of bone marrow stromal cells [21]. Clinically FD/MAS is characterized by polyostotic fibrous dysplasia (POFD), CALMS, and peripheral precocious puberty. On CT FD lesions have a characteristic ground-glass appearance, which are not observed radiographically in cherubism lesions [22]. In our case, FD/MAS was excluded based on the absence of this radiographic pattern and a negative genetic test for *GNAS* gene.

Central giant-cell granuloma (CGCG) is a rare non-odontogenic osteolytic disease that usually affects the jaw bones. The pathogenesis is unknown and CGCG is considered a benign asymptomatic swelling of the jaws, which histopathologically is characterized by multinucleated osteoclast-like giant cells, extravasated erythrocytes and a fibrous connective tissue stroma, which is similar to the histological profile of cherubism [5, 23]. Despite that, it has been reported that perivascular eosinophilic cufflike deposition is a specific histopathologic feature of cherubism [24]. In the present case, the patient not only had bilateral osteolytic lesions, but also had proteinaceous eosinophilic accumulation in the perivascular space effectively excluding CGCG from the differential diagnosis.

In conclusion, we report the co-occurrence of *NF1* and *SH3BP2* pathogenic variants in a case with NF1-related manifestations and cherubic phenotypic features. Since several nosological entities could share common clinical characteristics, genetic testing is a valuable tool in the pediatrician’s diagnostic armamentarium. Accurate diagnosis is of outmost importance, if we are to offer the best possible therapeutic guidance and reproductive options to these patients and/or their families.

## Data Availability

PubMed was used as a source of information and all additional data are available on request.
